# Plasma Kynurenine to Tryptophan Ratio Is Not Associated with Undernutrition in Adults but Reduced after Nutrition Intervention: Results from a Community-Based Study in Bangladesh

**DOI:** 10.3390/nu14091708

**Published:** 2022-04-20

**Authors:** Md. Amran Gazi, Md. Abdullah Siddique, Md. Ashraful Alam, Farzana Hossaini, Md. Mehedi Hasan, Shah Mohammad Fahim, Barbie Zaman Wahid, Md. Mamun Kabir, Subhasish Das, Mustafa Mahfuz, Tahmeed Ahmed

**Affiliations:** 1 Nutrition and Clinical Services Division, International Centre for Diarrhoeal Disease Research, Bangladesh (icddr,b), Dhaka 1212, Bangladesh; amran.gazi@icddrb.org (M.A.G.); mashraful@icddrb.org (M.A.A.); farzanasacky@gmail.com (F.H.); md.hasan@icddrb.org (M.M.H.); mohammad.fahim@icddrb.org (S.M.F.); barbiezwahid@gmail.com (B.Z.W.); subhasish.das@icddrb.org (S.D.); tahmeed@icddrb.org (T.A.); 2 Emerging Infections and Parasitology Laboratory, International Centre for Diarrhoeal Disease Research, Bangladesh (icddr,b), Dhaka 1212, Bangladesh; abdullah@icddrb.org (M.A.S.); mamunk@icddrb.org (M.M.K.); 3 Faculty of Medicine and Life Sciences, University of Tampere, 33100 Tampere, Finland; 4 Department of Global Health, University of Washington, Seattle, WA 98195, USA

**Keywords:** undernutrition, BMI, tryptophan, kynurenine, KT ratio, environmental enteric dysfunction, Bangladesh

## Abstract

Infections and persistent immunological activation are linked to increased kynurenine (KYN) and the KYN-to-Tryptophan (TRP) or KT ratio and may be critical factors in undernutrition. We sought to determine the association between the KT ratio and adult malnutrition, as well as investigate if nutritional supplementation had any influence on the decrease of the KT ratio. A total of 525 undernourished adults aged 18–45 years were recruited and provided a nutrition intervention for 60 feeding days. TRP and KYN concentrations were determined from plasma samples using LC-MS/MS. At baseline, the median (interquartile range (IQR)) TRP, KYN and KT ratios were 24.1 (17.6, 34.3) µmol/L, 0.76 (0.53, 1.18) µmol/L and 30.9 (24.5, 41.7), respectively. Following intervention, the median (IQR) KYN and KT ratios were significantly reduced to 0.713 (0.46, 1.12) µmol/L and 27.5 (21.3, 35.8). The KT ratio was found to be inversely linked with adult BMI (coefficient: −0.09; 95% CI: −0.18, 0.004; *p*-value = 0.06) but not statistically significant. Additionally, Plasma CRP was correlated positively, while LRP1 was inversely correlated with the KT ratio. Our data suggest that in Bangladeshi adults, the KT ratio is not related to the pathophysiology of malnutrition but correlated with inflammatory and anti-inflammatory biomarkers, and the ratio can be reduced by a nutrition intervention.

## 1. Introduction

Adult undernutrition, defined by a BMI (body mass index) of less than 18.5 kg/m^2^, has been linked to both immediate and long-term repercussions. Having a BMI <18.5 has been associated with poor IQ, unfavorable maternal reproductive outcomes, low adult wages and nutrition-related chronic diseases [1,2]. As a result of this phenomenon, the immune system is compromised, which increases the likelihood of infections [3]. Dietary diversity, psychiatric symptoms, gastrointestinal inflammation, altered gut health, and intestinal permeability are all factors that contribute to undernutrition [3]. Multiple enteric infections induce severe intestinal immune activation, increased intestinal permeability and prolonged systemic immunological activation, leading to Environmental Enteric Dysfunction (EED), also known as gut dysfunction [4,5]. EED is more prevalent among populations who have limited access to hygiene, sanitation and where multiple infections occur concurrently [5]. Consequently, the immunological response to these infections can take a variety of forms and be non-adaptive, leading to systemic inflammation [6]. Such exposures result in complete modifications in metabolic demands to an inherent alteration in the needs of energy and amino acids (AAs), resulting in undernutrition in the most severe infections [7]. Furthermore, disease-related undernutrition occurs as a result of decreased dietary intake, increased nutritional losses and malabsorption [8].

Tryptophan (TRP), a plant-obtained essential amino acid (EAA), is crucial for cellular respiration, neurotransmission and growth in humans [9]. Upon immune activation, the indoleamine 2,3-dioxygenase (IDO) catabolizes TRP in the vast majority of instances, which is triggered by the proinflammatory cytokine IFN-γ, and results in the formation of kynurenine (KYN) [10]. TRP reduction and the production of the immunomodulatory metabolite KYN may stifle T-cell growth and ultimately induce T-cell death [11]. Moreover, it has been shown elsewhere that dietary tryptophan consumption affects circulating levels of the metabolite KYN [12]. Thus far, the KYN–TRP (KT) ratio is a better indicator of tryptophan catabolism than kynurenine concentration alone as a biomarker of a systemic immune response.

The plasma KT ratio has been considered as a biomarker of systemic inflammation in conditions such as obesity, sepsis, inflammatory bowel disease, type 2 diabetes and immunodeficiency syndrome [13,14,15]. In addition, an increased KYN and KT ratio has been found to be associated with brain dysfunction [16,17]. In Peruvian and Tanzanian children, the KT ratio was also associated with growth inadequacies [18]. Moreover, in Malawian children, gut permeability was also reported to be positively correlated to serotonin/TRP and KT ratios. In a prior literature, we have shown that the KT ratio is significantly associated with stunting in Bangladeshi children, implicating the KT ratio in the pathophysiology of stunting [19,20]. It has also been proven that chicken eggs, cow’s milk and micronutrient supplementation exhibited growth-stimulating effects in Bangladeshi children [21]. In addition, nutritional interventions may aid in strengthening the immune system and lowering the chances of opportunistic infections [22]. However, the prospective association between malnutrition in adults and whether daily supplementation with chicken eggs, milk and essential micronutrients would have any effect on the reduction of KT ratio is yet to be elucidated. Therefore, we hypothesized that inflammation mediated by the KT pathway, which induces EAA deficiency, may be an essential factor for causing undernutrition in adults. The aim of this research was to assess the role of the KYN pathway in TRP metabolism in malnourished adults dwelling in a slum of Bangladesh and the effect of nutrition supplementation on the reduction of the KT ratio.

## 2. Materials and Methods

### 2.1. Study Design, Population and Ethical Considerations

We employed data from the Bangladesh Environmental Enteric Dysfunction (BEED) Study, which is still in progress, to conduct this research. This investigation was carried out among slum dwellers in Mirpur, Bangladesh. The protocol for this study has already been published elsewhere [23]. The study’s main goal was to validate non-invasive EED biomarkers and analyze the correlation between those biomarkers and stunting in children and undernutrition in adults. Undernourished adults between the ages of 18 and 45 were enlisted for a two-month intervention. The protocol contained micronutrient powder, an egg, 150 mL of whole milk, and nutritional coaching for 60 feeding days. Data were collected from 525 undernourished adults (<BMI 18.5 kg/m^2^) at the start of the study, with 512 participants available after the completion of the nutrition intervention. The icddr,b Institutional Review Board (IRB) authorized the research protocol (protocol no PR-16007). Before being enrolled in this study, individuals gave their informed written consent.

### 2.2. Data Collection and Biological Sample Collection and Storage

Trained field personnel used Standard Operating Procedures (SOPs) to measure anthropometry. Anthropometry data were obtained from all participants after enrolment by trained field staff. At the time of enrollment, every participant’s socioeconomic status was reported. Before and after the nutritional intervention, the subjects’ BMIs were examined. Samples (Stool and blood) were collected at the start and end of the dietary intervention. Whole blood was centrifuged for 10 min at 4000 revolutions per minute (rpm) to isolate the plasma. Stool and plasma aliquots were promptly frozen at −80 °C for assessment.

### 2.3. Assessment of TRP and KYN Concentrations Using LC-MS/MS and KT Ratio Determination

KYN, TRP and TRP-d5 reference compounds were acquired from Sigma (St. Louis, MO, USA), while KYN-d4 was procured from TLC pharmaceutical standards (Newmarket, ON, Canada) for this study. The mobile phase A contained a 9:75:16:0.3 (v) ratio of acetonitrile:tetrahydrofuran:25 mM ammonium formate:formic acid, while the mobile phase B contained a 20:80 ratio of acetonitrile:100 mM ammonium formate. A 50% (*v*/*v*) acetonitrile in water was utilized to prepare L-TRP and L-KYN standards at 1 mg/mL. TRP (10–1000 ng/mL) and KYN (2–200 ng/mL) were prepared using serial dilutions in water to create a calibration curve. KYN-d4 and TRP-d5 were used as internal standards, with concentrations of 50 ng/mL and 250 ng/mL, correspondingly.

Firstly, methanol/acetonitrile (1:1) was used to de-proteinize 20 μL of human plasma. Afterwards, 100 μL of the internal standard was added, proceeded by 10 min of vortexing and 10 min of centrifugation at 13,000 rpm. The filtrate was collected using a 0.2 m nylon filter. Subsequently, liquid chromatography with tandem mass spectrometry (LC-MS/MS) system was performed to analyze 10 μL of the samples. The system’s flow rate was set to 0.6 mL/minute, and each sample took 18.5 min to run. As a quality control measure, a standard solution was run randomly after every 10 samples. In this study, an LCMS-8050 (Shimadzu Corporation, Kyoto, Japan) was employed with an Intrada amino acid od 3 μm and a 100 × 3.0 mm column (Chrom Tech, Apple Valley, MN, USA). Essentially, we followed the procedure that our team had previously optimized for children [20]. The KT ratio was calculated by multiplying the quotient by 1000 and dividing the plasma concentration of KYN (mol/L) by the plasma concentration of TRP (mol/L) [20]. The KT ratio was utilized as a surrogate for IDO activity in this investigation.

### 2.4. Plasma and Stool Biomarkers Analysis Using ELISA

Using commercial enzyme-linked immunosorbent assay (ELISA) kits, alpha-1-acid glycoprotein (AGP) (Alpco, Salem, NH, USA), C-reactive protein (CRP) (Immundiagnostik, Bensheim, Germany), low-density lipoprotein receptor-related protein-1 (LRP1) (BIOMATIK, Wilmington, NC, USA), retinol-binding protein 4 (RBP4) (R&D Systems, Minneapolis, MN, USA) and ferritin (ORGENTEC Diagnostika GmbH, 55129 Mainz, Germany) were all assessed from the plasma samples. Concentrations of Zinc were tested from the plasma samples by the atomic absorption spectrometry method. Fecal biomarkers for neopterin (GenWay, San Diego, CA, USA), myeloperoxidase (Alpco, Salem, NH, USA), calprotectin (BUHLMANN fCAL, Schonenbuch, Switzerland), alpha-1-antitrypsin (AAT) (Biovendor, Chandler, NC, USA) and Reg1B (TechLab, Blacksburg, VA, USA) were assessed using ELISA, as per the manufacturer’s instruction manuals. The levels of each of the biomarkers were estimated using manufacturer-supplied standards. Stool was also tested for *Helicobacter pylori* (*H. pylori*) antigen (OXOID Limited, Hampshire, UK) using a qualitative ELISA test kit, with absorbance values of ≥0.15 being deemed positive and <0.15 being considered negative, following the kit’s manual.

### 2.5. Statistical Analyses

When the variables were categorical, socio-economic traits and demographic variables were characterized, employing frequencies with proportions. When quantitative variables were symmetrically distributed, means and standard deviations (SD) were presented, but when quantitative variables were asymmetrically distributed, medians and interquartile ranges (IQRs) were utilized. Differences in the KT ratio, TRP and KYN were investigated using a sign test, both before and after the intervention, with the variables being non-parametric and continuous.

At both the baseline and endpoint, correlations between the KYN and KT ratio and EED biomarkers and inflammation as well as other relevant biomarkers were investigated using Spearman’s correlation tests. The association between the KT ratio and BMI was evaluated using a multivariable linear regression with generalized estimating equations (GEE) while adjusted for sex, age, hygiene, maternal height, family income, micronutrients and inflammatory and fecal biomarkers. The concentrations of biomarkers were log-transformed prior to the analysis. Firstly, the unadjusted effect of each explanatory variable on the outcome variable was determined using a bivariate analysis. Covariates were then chosen for multivariable models if their association with the outcome had a significance of <0.2. Finally, GEEs were used to perform multivariable analyses to examine the associations after adjusting for potential confounders. The significance level was set at *p* < 0.05. The unstructured correlation matrix was employed in the GEE model, along with the Gaussian family and the identity as the link function. Using the independent model criteria (QIC) value, the correlation matrix was chosen based on the lowest quasi-likelihood. The GEE methodology allowed for the establishment of a working correlation matrix for the within-subject correlation of recurrent responses from the same respondents across time, resulting in more impartial and effective regression parameters. R version 3.5.1 (https://www.r-project.org, accessed on 30 November 2021) was used to conduct all statistical analyses.

## 3. Results

### 3.1. Socio-Demographic and Clinical Characteristics

A total of 525 malnourished adult participants were enrolled in this study, and 512 were available after the nutrition intervention. Table 1 depicts the baseline and endline characteristics of the participants.

### 3.2. Plasma Concentrations of TRP and KYN and the KT Ratio in Study Participants

**Figure 1:**
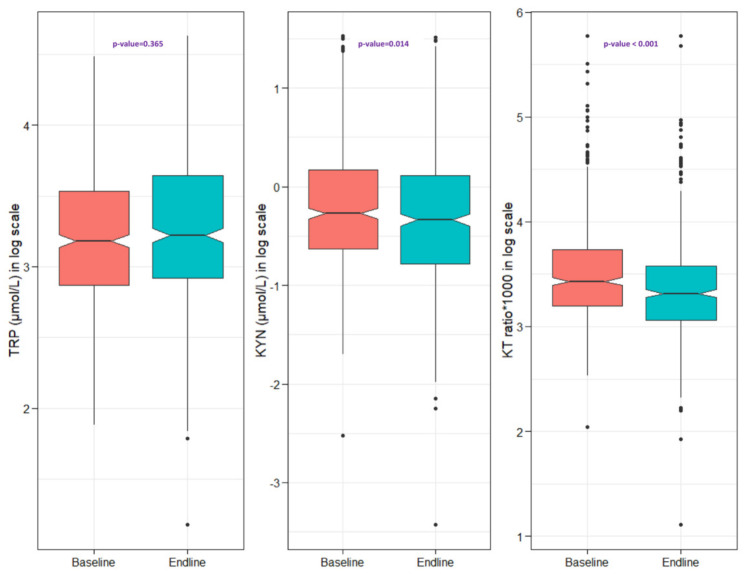
Baseline and endline concentrations of plasma tryptophan and kynurenine and the KT ratio in malnourished adults.

Figure 1 depicts the KT ratio and TRP and KYN concentrations at baseline, as well as comparisons at the end of the study. The median concentration of KYN had decreased significantly from 0.76 (0.53, 1.18) µmol/L at baseline to 0.71 (0.46, 1.12) µmol/L at endline, and the KT ratio had declined significantly from 30.9 (24.5, 41.7) at baseline to 27.5 (21.3, 35.8) at endline. There were no considerable differences between the baseline and endline TRP levels, which were 24.1 (17.6, 34.3) µmol/L at baseline and 25.1 (18.5, 38.3) µmol/L at the end of the study.

### 3.3. Correlation of TRP, KYN and KT Ratio with Fecal and Plasma Biomarkers in All Individuals

The Spearman’s rank correlation coefficient was employed to analyze the relationships between different markers both at baseline and endline in this study. At the endline of the study, there was a significant positive correlation between the KT ratio and CRP (correlation coefficient: 0.11); however, a significant negative correlation was found between the KT ratio and LRP1 at endline (correlation coefficient: −0.17). KYN and LRP1 also had a significant positive correlation at baseline (correlation coefficient: 0.14), while KYN and AAT had a significant positive correlation at endline (correlation coefficient: 0.13). At the baseline, there was no correlation between the KT ratio and any of the plasma or fecal biomarkers (Supplemental Tables S1 and S2).

### 3.4. Association of the KT Ratio and Related Variables with BMI

The investigation was longitudinal, with pre- and post-intervention samples and anthropometric data gathered from the same individuals. To examine the relationship between biomarkers and BMI, all subjects were considered at both the baseline and endline. Using GEE, Table 2 illustrates the relationship between the KT ratio and other variables and BMI. After adjusting for relevant confounders, the KT ratio was shown to be negatively associated with BMI (coefficient: −0.09; 95% CI: −0.18, 0.004; *p*-value = 0.06), although it was not statistically significant. The negative coefficient (−0.09) indicates that for every one unit increase in the KT ratio, the mean drop in BMI was −0.09 units. LRP1 showed a significant positive relationship with BMI (coefficient: 0.34; 95% CI: 0.20, 0.49; *p*-value < 0.001), while ferritin showed a significant negative association with BMI (coefficient: −0.13; 95% CI: −0.19, −0.07; *p*-value < 0.001) in adults.

## 4. Discussion

The elevated dangers of reduced productivity, lower IQ and impaired economic development, as well as increased morbidity and mortality linked to undernutrition, necessitate more research into the origin, prevention and early treatment of this condition in adults [24]. The increased mortality rate associated with undernutrition may be due to impaired immunological dysfunction resulting in repeated infections, the underlying mechanisms for which are still unknown. Although not statistically significant, the present study demonstrated a negative association between the KT ratio and undernutrition in Bangladeshi adults. However, we observed that both KYN and the KT ratio were significantly reduced after a daily dietary supplementation with 150 mL of milk, 1 egg and 1 RDA of multiple micronutrient powder for 60 feeding days. Additionally, in the multivariable model, we found that plasma LRP1 was positively, while ferritin was negatively, associated with adult BMI. 

The kynurenine pathway accounts for more than 95% of tryptophan metabolism, with the first and rate-limiting step catalyzed by either the enzyme tryptophan 2,3-dioxygenase (TDO) in the liver or the ubiquitous IDO1 [25]. TRP metabolism is transferred from hepatic to extrahepatic tissues in settings of increasing inflammation [26]. For instance, low TRP levels have been linked to cachexia or weight loss in neoplasia patients [27]. Increased TRP catabolism via the KYN route may cause this EAA to be shifted from protein synthesis, resulting in muscle breakdown and weight loss. Because of the conflict between protein growth and utilization by the immune system, AA partitioning occurs during immune system activation. When the supply of EAAs like cysteine, methionine, threonine and TRP is limited, the immune system competes for the AAs more fiercely, which might be a cause of undernutrition in adults. A study revealed the plasma concentration of the KT ratio to be associated with growth deficiencies among children coming from resource- constrained backgrounds in Peru and Tanzania [18]. Furthermore, our previous study exhibited a positive correlation between stunting in children from low socio-economic backgrounds and the KT ratio [20]. Although not statistically significant, our present study showed a negative association between BMI among malnourished adults and the KT ratio. However, a previous study conducted among TB patients showed a significant inverse association with the plasma KT ratio and BMI in adults [28]. These findings suggest that children may be more susceptible to infectious agents and that their immune system happens to be more vulnerable, which may, in turn, result in activation of immune system biomarkers and subsequent malnutrition in children. Moreover, low socioeconomic status, enteric infections and a variety of dietary factors that affect microbiome changes may all be linked to the undernutrition. Malnutrition can make a person more prone to infections, and the effects of recurrent enteric infections on malnutrition are a significant contributing factor [29]. Enteric infections are more prevalent in people who are undernourished, and consequently, the infection could alter the TRP pathway and result in immune dysfunction [29]. Besides this, weight loss, reduced immunity, mucosal injury, pathogen invasion and impaired growth and development are all symptoms of inadequate nutritional intake [30]. However, we did not include pathogen data or dietary factors in this study and were not able to make a conclusion about how they influence the KT ratio and undernutrition in adults.

Nutritional deficiency has long been known to severely impair the functioning of the immune system. The properties of nutrition and phytochemicals in modulating immune function have major implications for inflammation-mediated conditions [31]. An earlier survey demonstrated that food containing high macro and micronutrients, such as eggs, yogurt and cheese, contain excellent immunomodulatory, anti-inflammatory, antioxidant and antiviral activities [32]. A previous study also demonstrated that malnourished children who had undergone a daily dietary supplementation with 150 mL of milk, 1 egg and micronutrient powder for 3 months exhibited improved length-for-age scores [21]. The study also described the growth stimulating effects of egg and milk, leading to an increase in linear growth in children. These previous results also emphasized and were in line with our present research outputs, where we observed that daily supplementation with protein-based food and micronutrients could reduce the KT ratio in adults.

Our results also exhibited that the KT ratio positively correlates with CRP at the endline. CRP is a biomarker and measures systemic inflammation, and our study finding is in line with a previous report that showed that patients with flaky paint dermatosis had lowered TRP levels and enhanced activity of IDO because of increased CRP levels [33]. In a previous study, IDO activity was shown to correlate positively with the marker of atherosclerosis, especially in young female adults [34]. Moreover, patients with coronary heart disease showed lower TRP levels and higher IDO activity [35]. The higher IDO activity and KT ratio in chronic kidney disease have also been linked with high-sensitive-CRP and soluble TNF receptor-I, regardless of age, body weight or serum creatinine [36]. Additionally, we found a significant negative correlation with the KT ratio and lipoprotein receptor-related protein-1 (LRP1) after the nutrition intervention. LRP1 is an endocytic receptor that plays an essential role in phagocytosis, necessary for the removal of infectious agents. Previous studies have shown that LRP1 is negatively correlated with intestinal inflammatory biomarkers in Bangladeshi children, while an increased level of LRP1 was associated with an increased BMI in Bangladeshi adults [37,38]. In this study, in the multivariable model analysis, we also found LRP1 to be positively associated with BMI, which strengthens the previous findings. Our current analysis additionally found a significant negative association of ferritin with adult BMI. Ferritin is an acute phase reactant and a marker of chronic inflammation, whereby its levels usually go up or down depending on how much iron is in the body [39]. However, ferritin also increases with inflammation, irrespective of iron levels. Inflammation is a common entity in undernutrition, and that might be the cause of a lower BMI with increasing levels of ferritin in this study. Findings from a previous study imply that serum ferritin is both a marker of iron status and an indicator of inflammation and/or malnutrition in dialysis patients [39].

There are a few limitations to this study. Firstly, there was no control group to assess if the supplementation caused the reduction of KYN and the KYN–TRP ratio. To verify the supplementing effect, a control group (who did not receive supplements) is required for comparison. Secondly, our study showed that the KT ratio positively correlated with biomarkers of systemic inflammation. A correlation does not necessarily confirm a causal relationship. In addition to this, we did not measure IDO activity directly but by estimating the KT ratio, although it has been shown to accurately assess the respective enzyme activities in a previous study. Increased expressions of immunomodulatory indicators such as interferon-gamma, interleukin-6, interleukin-10, CD4 and CD8, as well as increased TRP catabolism by IDO, have also been linked to increased TRP catabolism by IDO [40]. However, none of the cytokines described above were tested in our research. Moreover, in the current study, we did not evaluate the cost effectiveness of testing the KT ratio for malnutrition and whether this indicator is realistically feasible for people without money. Testing the KT ratio using a high throughput LC-MS/MS technique is currently limited by the need for highly trained personnel and resources, but like many other tools, it could potentially be translated to simpler methods for near-care testing in the future. This study’s strength is the inclusion of a significantly larger sample size. Furthermore, all the biomarkers were examined in a single batch to eliminate batch effects and inter-tester variation. Finally, the current study only found a positive association between inflammatory biomarkers and the KT ratio, but the underlying mechanism of this association is yet to be addressed.

In summary, our data suggested that the KT ratio was negatively associated with BMI in malnourished adults, although the association was not statistically significant. In addition, the KT ratio was correlated with biomarkers of inflammation and could be lowered through nutrition intervention. However, fundamental concerns about the underlying mechanism of how KYN or KT ratios are lowered in the context of undernutrition among Bangladeshi adults remain unanswered and might be investigated further in prospective research.

## Figures and Tables

**Table 1:** Baseline characteristics of the malnourished adults at enrolment and at the end of the nutrition intervention.

Indicators	Baseline (*n* = 525)	Endline (*n* = 512)	Overall (*n* = 1037)	*p*-Value
Age in years, Mean (SD)	23.8 (6.83)	-		-
Female gender, *n* (%)	382 (72.8)	-		-
Weight in kg, Mean (SD)	41.4 (4.79)	42.3 (5.03)	41.8 (4.93)	<0.001
Height in cm, Mean (SD)	155 (8.04)	155 (8.13)	155 (8.08)	0.318
BMI in kg/m^2^, Mean (SD)	17.3 (0.83)	17.6 (1.13)	17.4 (1.01)	<0.001
TRP (µmol/L), Median (Q1, Q3)	24.1 (17.6, 34.3)	25.1 (18.5, 38.3)	24.4 (18.1, 36.2)	0.365
KYN (µmol/L), Median (Q1, Q3)	0.76 (0.53, 1.18)	0.71 (0.46, 1.12)	0.73 (0.49, 1.15)	0.014
KT ratio, Median (Q1, Q3)	30.9 (24.5, 41.7)	27.5 (21.3, 35.8)	29.3 (22.7, 38.8)	<0.001
Infection with *H. pylori*, *n* (%)	421 (80.3)	403 (80.8)	824 (80.5)	0.866
NEO (nmol/L), Median (Q1, Q3)	1010 (392, 1830)	1070 (396, 1850)	1040 (392, 1840)	0.530
MPO (ng/mL), Median (Q1, Q3)	737 (355, 1460)	664 (383, 1480)	711 (366, 1460)	0.369
AAT (mg/g), Median (Q1, Q3)	0.14 (0.06, 0.30)	0.16 (0.07, 0.30)	0.15 (0.06, 0.30)	0.684
CRP (mg/L), Median (Q1, Q3)	0.65 (0.23, 1.57)	0.63 (0.24, 1.8)	0.64 (0.23, 1.66)	0.719
LRP1 (ng/mL), Median (Q1, Q3)	807 (629, 1060)	873 (619, 1200)	832 (621, 1120)	0.015
Income in USD, Median (Q1, Q3)	179 (122, 239)	-		-
Improved sanitation, *n* (%)	377 (71.8)	-		-

Abbreviations used: BMI, body mass index; TRP, tryptophan; KYN, kynurenine; KT, kynurenine-to-tryptophan; AAT, alpha-1 antitrypsin; MPO, myeloperoxidase; NEO, neopterin; CRP, C-reactive protein; LRP, low-density lipoprotein receptor-related protein.

**Table 2:** Association of the KT ratio and other variables with body mass index using a generalized estimating equation at baseline and endline (*n* = 525).

Variables	Unadjusted β (95% CI)	*p*-Value	Adjusted β (95% CI)	*p*-Value
Age in years	0.01 (0, 0.02)	0.15	0.01 (0, 0.02)	0.04
Sex (Female)	0.09 (−0.09, 0.27)	0.34	0.02 (−0.17, 0.22)	0.80
KT ratio	−0.11 (−0.21, −0.02)	0.02	−0.09 (−0.18, 0)	0.06
AAT	0.00 (−0.04, 0.04)	0.90		
MPO	−0.03 (−0.06, 0.01)	0.12	−0.02 (−0.06, 0.01)	0.17
NEO	0.00 (−0.03, 0.04)	0.83		
Calprotectin	−0.03 (−0.07, 0.01)	0.17	−0.01 (−0.05, 0.03)	0.60
Reg1B	−0.01 (−0.03, 0.01)	0.48		
Infection with *H. pylori*	−0.07 (−0.22, 0.09)	0.40		
AGP	0.12 (0.01, 0.22)	0.04	0.09 (−0.02, 0.2)	0.09
CRP	−0.01 (−0.04, 0.02)	0.55		
Ferritin	−0.13 (−0.19, −0.07)	<0.001	−0.13 (−0.19, −0.07)	<0.001
RBP4	0.07 (−0.08, 0.21)	0.38		
LRP1	0.38 (0.23, 0.52)	<0.001	0.34 (0.2, 0.49)	<0.001
zinc	0.15 (−0.15, 0.45)	0.34		
Family income ($100 increase)	0.00 (0.00, 0.00)	0.20	0.00 (0.00, 0.00)	0.09
Improved sanitation	0.17 (−0.01, 0.35)	0.06	0.14 (−0.03, 0.32)	0.10

Abbreviations used: KT, kynurenine-to-tryptophan; AAT, alpha-1 antitrypsin; MPO, myeloperoxidase; NEO, neopterin; Reg1B, regenerating family member 1 beta; CRP, C-reactive protein; AGP, Alpha-1-acid glycoprotein; RBP, retinol-binding protein; LRP, low-density lipoprotein receptor-related protein.

## Data Availability

All relevant data are within the manuscript.

## Supplementary Materials

The following supporting information can be downloaded at https://www.mdpi.com/article/10.3390/nu14091708/s1. Table S1: Spearman rank correlation between different indicators at baseline; Table S2: Spearman rank correlation between different indicators at endline.
