# Supplementary material for: Plasma Kynurenine to Tryptophan Ratio Is Not Associated with Undernutrition in Adults but Reduced after Nutrition Intervention: Results from a Community-Based Study in Bangladesh
Source: Nutrients. 2022 Apr 20;14(9):1708. doi: 10.3390/nu14091708 (PMC9104876; doi:10.3390/nu14091708)
Supplement: Supplementary file 1 [file nutrients-14-01708-s001.zip › nutrients-1657992-supplementary.pdf]

Table S1: Spearman rank correlation between different indicators at baseline

Correlation among biomarkers at baseline

	Tryptophan	Kynurenine	KT ratio	MPO	NEO	AAT	CRP	Ferritin	LRP1
Tryptophan	1								
Kynurenine	0.57*	1							
KT ratio	0.07	0.68*	1						
MPO	0.006	0.06	0.05	1					
NEO	0.09*	0.06	0.01	0.03	1				
AAT	0.001	0.0004	0.01	0.17*	0.03	1			
CRP	0.07	0.01	0.03	0.07	0.03	0.01	1		
Ferritin	0.02	0.07	0.04	0.03	0.006	0.07	0.22*	1	
LRP1	0.15*	0.13*	0.001	0.04	0.03	0.02	0.07	0.07	1

*P<0.05

Table S2: Spearman rank correlation between different indicators at endline

	Tryptophan	Kynurenine	KT ratio	MPO	NEO	AAT	CRP	Ferritin	LRP1
Tryptophan	1								
Kynurenine	0.63*	1							
KT ratio	0.0001	0.63*	1						
MPO	-0.07	-0.08	-0.06	1					
NEO	0.02	0.02	0.02	-0.04	1				
AAT	0.13*	0.13*	0.08	0.03	0.02	1			
CRP	-0.04	0.04	0.11*	0.03	-0.03	0.05	1		
Ferritin	0.0003	-0.02	-0.03	-0.01	0.05	0.08	0.17*	1	
LRP1	-0.16	-0.21	-0.17*	0.08	0.05	-0.07	0.08	0.09	1

*P<0.05
